# Applications and challenges of photoacoustic technology in internal medicine

**DOI:** 10.1515/jtim-2026-0051

**Published:** 2026-06-13

**Authors:** Junyu Zhou, Yuting Fu, Xunbin Wei

**Affiliations:** Institute of Advanced Clinical Medicine, Peking University, Beijing, China; Institute of Medical Technology, Peking University Health Science Center, Beijing, China; Department of Biomedical Engineering, Peking University, Beijing, China; Key Laboratory of Carcinogenesis and Translational Research (Ministry of Education/Beijing), Peking University Cancer Hospital and Institute, Beijing, China

## Introduction

In contemporary medicine, the diagnosis and treatment of internal diseases are evolving toward higher resolution, enhanced functionality, and reduced invasiveness. Common chronic diseases, exemplified by cardiovascular disease and diabetes, have created unprecedented demands for precision imaging and dynamic monitoring. Photoacoustic (PA) technology, an emerging multifunctional technique that combines the advantages of optics and ultrasound, is no longer limited to traditional imaging applications but has gradually expanded to multiple dimensions including therapeutic assistance, drug delivery, and pathological microenvironment monitoring. This article focuses on the clinical potential and translational challenges of PA technology in the field of internal medicine, and attempts to provide pathway recommendations for its interdisciplinary integration and development.

## Principles of photoacoustic technonogy and its medical application advantages

PA Technology combines the dual advantages of optical resolution and acoustic penetration depth, possessing exceptional cross-scale analytical capabilities and functional imaging potential. Its core principle is based on the PA effect. When pulsed laser light of specific wavelengths irradiates tissue, optical absorbers within the tissue, such as hemoglobin, lipids, water, or functional molecular probes, undergo nanosecond-level thermal expansion, subsequently generating broadband ultrasonic waves.^[1]^ By receiving these signals through high-frequency ultrasonic transducers and combining multidimensional algorithms to reconstruct tissue detail information, simultaneous characterization of tissue structure and function can be achieved. Particularly in clinical scenarios requiring frequent imaging for chronic disease monitoring, PA technology offers clear advantages, including non-ionizing, radiation-free operation, real-time capability, high sensitivity to hemoglobin and oxygenation changes, and suitability for repetitive, longitudinal evaluations without cumulative risk to patients.

Compared with traditional imaging modalities, PA technology demonstrate significant advantages in medical scenarios. (1) Non-invasive real-time blood oxygen quantification, PA imaging can precisely differentiate between oxygenated and deoxygenated hemoglobin, enabling tissue-level oxygen supply assessment, particularly suitable for oxygen metabolism disorder-related diseases such as myocardial ischemia, chronic kidney disease, and diabetic foot. (2) Quantitative lipid and fiber imaging, sensitive to light-absorbing components such as lipids and cholesterol, applicable for visualization of lipid deposition in patients with hepatic steatosis, atherosclerosis, and metabolic syndrome. (3) Lesion metabolic activity monitoring, combined with molecular probes, such as pH and H_2_O_2_-sensitive dyes, enables multidimensional dynamic monitoring of lesion inflammation, oxidative stress, and metabolic states, demonstrating high potential in autoimmune diseases and tumor immunity-related conditions. (4) Deep tissue penetration with maintained resolution, compared to pure optical methods such as Optical coherence tomography (OCT) or reflectance optical imaging, PA signals can penetrate depths of several centimeters while maintaining submillimeter resolution, suitable for detecting deep muscle and peripulmonary lesions. (5) Radiation-free, repeatable monitoring, particularly suitable for long-term, frequent follow-up monitoring in chronic disease populations, such as hepatic fibrosis progression in chronic hepatitis patients and dynamic assessment of renal perfusion in Chronic kidney disease (CKD) patients. (6) Ultrasound integration facilitating clinical implementation, under appropriate ultrasonic probe parameters, PA can share probe systems with existing ultrasound equipment, requiring only minimal physician training for rapid adoption, reducing the learning curve. (7) Compatibility with wearable and bedside monitoring devices, portable PA devices can already be applied for diabetic foot perfusion assessment and postoperative microcirculation recovery monitoring, promoting home-based and mobile implementation of PA technology in chronic disease management. These advantages make PA technology not merely an imaging tool that sees clearly, but rather a functional diagnostic and therapeutic platform that understands and sees accurately. Its cross-modal, functional, and intelligent characteristics precisely match the high-level demands of internal medicine clinical practice for precise, dynamic, and non-invasive monitoring.^[2]^

## Key applications of photoacoustic technology in internal medicine

**Figure 1:**
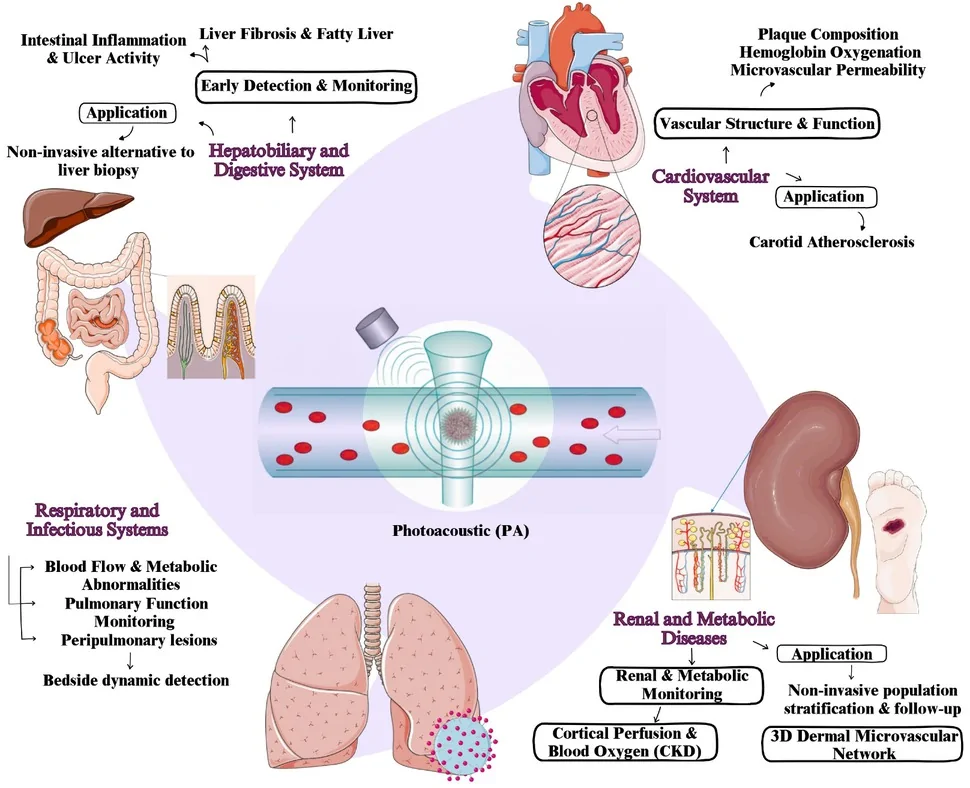
Clinical applications of emerging photoacoustic technology for internal medicine diagnosis and treatment. Images provided by Servier Medical Art (https://smart.servier.com), licensed under CC BY 4.0 (https://creativecommons.org/licenses/by/4.0/).

Traditional internal medicine disease diagnosis and treatment require techniques such as laboratory testing, imaging examinations, pharmacological therapy, and interventional treatment. PA technology utilizes endogenous contrast from human HbO_2_/Hb, lipids, and other substances to provide structural-functional-metabolic information at millimeter-to-micrometer resolution, giving PA technology increasing diagnostic and therapeutic advantages in cardiovascular, hepatobiliary, renal, respiratory, and infectious diseases (Figure 1).

### Cardiovascular system

PA technology can be used to assess vascular structure and functional status, including plaque composition identification, hemoglobin oxygenation changes, and microvascular permeability. *In vivo* PA technology for carotid atherosclerosis has already demonstrated good prospects in detecting intraplaque hemorrhage and specific lipid components. In carotid atherosclerosis, *in vivo* PA technology has demonstrated promising prospects in detecting intraplaque hemorrhage and specific lipid components, as evidenced by ex vivo studies showing strong qualitative alignments between unmixed hemoglobin signals and histological markers of fibrin across Near-Infrared (NIR) wavelengths (850–1250 nm).^[3]^

### Hepatobiliary and digestive system

In early detection of liver fibrosis and fatty liver disease, PAs can quantify lipid accumulation and oxygen supply conditions, and may even potentially serve as a non-invasive alternative to liver biopsy. PA endoscopy can also provide layered structural and vascular functional information for evaluating intestinal inflammation and ulcer activity, offering new assessment tools for inflammatory bowel diseases. Spectral PA imaging in animal models has correlated well with histological fibrosis grades, enabling quantification of collagen deposition, steatosis, and hemodynamic changes that align with METAVIR-like staging systems.^[4]^

### Renal and metabolic diseases

Chronic kidney disease patients often rely on renal function indicators and imaging follow-ups. PA technology can assist in detecting cortical perfusion and blood oxygen levels, providing support for alternative non-invasive monitoring methods. Additionally, in peripheral circulation disorders such as diabetic foot, PAs can provide three-dimensional details and functional information of the full-thickness dermal microvascular network, small vessel number (< 40 μm diameter), large vessel number (> 40 μm), total vessel number, total blood volume (as voxel occupancy ratio), epidermal thickness, and epidermal signal density,^[5]^ assisting clinical differentiation of different stages of diabetes and reflecting complication risks, suitable for noninvasive population stratification and follow-up.^[6]^

### Respiratory and infectious systems

PA can be used to detect blood flow and metabolic abnormalities in peripulmonary lesions, particularly suitable for bedside dynamic detection. Research has also explored its role in monitoring pulmonary function during coronavirus disease 2019 (COVID-19) recovery.^[7]^

## Beyond imaging: Functional extensions of photoacoustic technology

In recent years, PA technology has gradually achieved a transformation from seeing clearly to understanding and intervening in internal medicine clinical practice. Representative applications include: (1) combined photothermal therapy, providing localization and energy feedback monitoring for focal lesions ^[8]^; (2) *in vivo* liquid biopsy, with *in vivo* PA flow cytometry enabling noninvasive detection of circulating tumor cells within patients, assisting disease monitoring and metastasis risk assessment^[9]^; (3) drug release monitoring, achieving precise tracking through nanocarriers in PA imaging^[10]^; (4) microenvironment regulation, such as detecting tissue pH and oxidative stress indicators to achieve inflammatory activity assessment.^[10]^ These functional advances are expected to achieve important breakthroughs in early intervention of autoimmune diseases, tumor metastasis, metabolic syndrome, and other conditions.

## Bottlenecks and opportunities in clincial translation

Despite rapid development of PA technology, widespread clinical application remains constrained by multiple barriers. Imaging depth limitations, particularly in obese patients or abdominal organ imaging. Developing PA imaging technology based on deep penetration wavelengths (such as near-infrared-II region) and novel transducers to enhance imaging depth in abdominal and obese patients can effectively address these technical issues. Inconsistent equipment standards, with significant variations among commercially available devices and lack of unified parameters and image quality assessment systems. Establishing national-level PA image quality control standards and unified imaging protocols to promote interoperability between different devices and data can effectively resolve this problem. Laser safety concerns, with irradiation limited to American National Standards Institute (ANSI) safety standards and contraindications including photosensitive conditions. Implementing optimized laser parameters, strict adherence to safety guidelines, and routine patient screening can effectively manage these risks. Significant data gaps, with insufficient clinical multicenter samples and lack of real-world validation data. Leveraging big data platforms and AI automatic annotation algorithms to construct multicenter real-world datasets can enhance model generalization capability. To promote the clinical translation of PA technology-related devices, there is a need to include PA devices in China’s Medical Device Classification Catalog and Special Review Procedures for Innovative Medical Devices. Internationally, similar regulatory pathways exist through Food and Drug Administration (FDA) premarket approval in the USA andConformité Européenne (CE) marking in Europe, with one PA device currently holding FDA approval for the evaluation of breast abnormalities.

## Conclusion and future prospects

PA technology has advanced from laboratory to the clinical frontier. Its unique advantages in functional imaging, multimodal fusion, and non-invasive capabilities particularly align with current internal medicine demands for non-invasive, intelligent, and precise tools. Future development should focus on practicality, standardization, and interpretability as core principles, promoting deep collaboration from device development and algorithm optimization to clinical co-construction. Through interdisciplinary cooperation and experimental research support, PA technology is expected to truly become the third eye of internal medicine physicians, playing unique value in chronic disease management, bedside decision-making, and precision treatment.
